# Dynamic Neurocognitive Adaptation: a follow‐up exposome investigation in aging

**DOI:** 10.1002/alz70858_106986

**Published:** 2025-12-25

**Authors:** Filippo Cieri, Chad L. Cross, Giulia Di Francesco, Jessica ZK Caldwell

**Affiliations:** ^1^ Cleveland Clinic, Las Vegas, NV, USA; ^2^ University of Nevada Las Vegas, Las Vegas, NV, USA; ^3^ Pescara Hospital, Pescara, Pescara, Italy; ^4^ Nevada Exploratory Alzheimer's Disease Research Center, Las Vegas, NV, USA; ^5^ Cleveland Clinic Lou Ruvo Center for Brain Health, Las Vegas, NV, USA

## Abstract

**Background:**

45% of dementia cases are preventable. Investigation of protective factors through validated tools could help the imbalance in measurement precision between biogenetic and environmental factors contributing to public health benefits. The *dynamic Neurocognitive Adaptation* (dNA) Scale is a validated tool to explore protective factors in AD.

**Method:**

Correlative associations among cognitive, physical, creative, and social domains were assessed using distance correlations. We examined domains across the seven time periods using RM‐ANOVA with Greenhouse‐Geisser sphericity. We calculated within‐subjects comparisons for time and all interactions among time, sex, and education. We examined between‐subjects factors for sex, education, and their interaction. From these models, we constructed visualizations of estimated marginal means against time to assess potential patterns of interest, using a significance threshold of 0.05.

**Result:**

The physical and creative domains were significantly correlated (*p* < 0.001). The social domain was correlated with both the physical (*p* < 0.001) and creative (*p* = 0.047) domains among females but not males. In cognitive domain, there was an increase in scores as a function of time (*p* < 0.001), with women having higher scores than men (*p* = 0.024). In the physical domain, there was an increase from childhood to adolescence followed by a decrease as a function of time, with men showing higher scores (*p* = 0.011). In creative domain, there was an increase from childhood to adolescence followed by a decline through senior age and a drop to old age (*p* < 0.001). In the social domain, there was an increase from childhood to adolescence followed by a decline through the senior years and a steep increase in old age. In all dimensions the scores were higher as a function of education.

**Conclusion:**

Our scale investigates protective factors in AD, providing an opportunity to further investigate the exposome in aging.

